# Establishment and internal-external validation of a 28-day mortality prediction model for septic shock patients with left ventricular systolic dysfunction

**DOI:** 10.3389/fmed.2026.1841230

**Published:** 2026-06-30

**Authors:** Jia Lin, Xiaojia Wang, Kai Chen, Yafang Liu, Yizhuo Zhang, Tianmiao Xu, Xiaojuan Yang

**Affiliations:** 1 The First School of Clinical Medicine, Ningxia Medical University, Yinchuan, China; 2 General Hospital of Ningxia Medical University, Yinchuan, China

**Keywords:** APACHE II score, LVEF, LVSD, nomogram, prognostic prediction model, septic shock

## Abstract

**Background:**

Septic shock remains a major cause of death in the ICU with high 28-day mortality, and concomitant left ventricular systolic dysfunction (LVSD) further worsens patient outcomes, while existing prediction models lack adequate specificity. This study aimed to establish and perform internal-external validation of a 28-day mortality prediction model specifically for septic shock patients with LVSD as a key risk factor.

**Methods:**

This retrospective multicenter cohort study enrolled 226 septic shock patients from September 2021 to October 2024 as the training cohort and 132 patients from November 2024 to December 2025 as the validation cohort, from ICUs of General Hospital and Cardiovascular Hospital of Ningxia Medical University. Baseline LVEF was measured at admission, with LVSD defined as LVEF < 50% or > 70%. Baseline data, clinical variables, myocardial biomarkers, and 28-day mortality were collected. Univariate and multivariate logistic regression identified independent predictors. A predictive nomogram was constructed and assessed using ROC, calibration curves, and DCA. Internal validation was performed via 1,000 bootstrap resamples, followed by external validation in the validation cohort.

**Results:**

Independent predictors of 28-day mortality in patients with septic shock were: left ventricular systolic dysfunction, decreased pH, atrial arrhythmia, dopamine use, and reduced PaO_2_/FiO_2_. Based on these predictors, a predictive model for 28-day mortality in patients with septic shock was constructed, and a nomogram was developed. The area under the ROC curve (AUC) of the model in the training cohort was 0.767 (95% CI: 0.703–0.831). After internal validation via Bootstrap sampling, the mean AUC was 0.779 (95% CI: 0.713–0.841). The calibration curve approached the ideal line (Hosmer–Lemeshow test *P* = 0.476), and the DCA indicated clinically net benefit within the 0.05–0.85 probability threshold range. External validation confirmed the reliability of the predictive model. Further comparison revealed that the predictive performance of this model was significantly superior to that of the APACHE II score for predicting mortality (0.767 vs. 0.652, *P* = 0.006). This nomogram has been converted into a web-based dynamic nomogram calculator available for free public use (https://linjia.shinyapps.io/dynnomapp/).

**Conclusion:**

The 28-day mortality prediction model demonstrated excellent discriminative power, stability, and clinical applicability.

## 1 Introduction

Sepsis is a life-threatening organ dysfunction caused by dysregulation of the host response to infection. As one of the leading causes of death among patients in intensive care units (ICUs) worldwide, it has become a major public health issue posing a serious threat to human health (1). septic shock, a critical subtype of sepsis, carries an even heavier epidemiological burden, with a global incidence rate of approximately (50–70)/100,000 population and a hospital mortality rate exceeding 40% (2, 3). Characterized by rapid disease progression and a narrow therapeutic window, it represents a major clinical challenge requiring urgent resolution in critical care medicine (4, 5).

The heart is one of the core target organs most susceptible to involvement during sepsis and septic shock, with cardiac dysfunction closely linked to poor patient outcomes. Among these, left ventricular systolic dysfunction (LVSD) represents the most common myocardial abnormality in patients with septic shock, accurately assessed through echocardiographic indicators such as left ventricular ejection fraction (LVEF). Studies have confirmed that the incidence of LVSD in patients with septic shock can reach 20–60%, and patients with this dysfunction often face higher mortality risks and poorer outcomes (6, 7). Not only conventional cardiac depression with LVEF < 50% but also the hyperdynamic circulatory state with LVEF > 70% are categorized as LVSD in the present study (8). Pathophysiologically, the hyperdynamic state reflects compensatory overactivation of cardiac contractility driven by overwhelming inflammatory stress and neurohumoral regulation in septic shock, which essentially represents an abnormal adaptive response rather than normal cardiac function (9). Therefore, this study incorporates these two abnormal states of myocardial contraction function into the LVSD category, aiming to improve the accuracy of identifying cardiac dysfunction in patients with septic shock and further optimize the prognostic evaluation model for septic shock patients based on cardiac dysfunction.

The mortality rate of patients with septic shock remains high, which may be related to the inadequacies of clinically available prognostic assessment and risk identification tools, hindering early detection and proactive intervention of the disease. Although traditional sepsis prognostic scoring systems, such as the APACHE II score (Acute Physiology and Chronic Health Evaluation II), have been widely used in clinical practice, these scoring systems often do not systematically incorporate indicators related to cardiac function, particularly lacking integration of the left ventricular systolic function, which is an important factor affecting prognosis (10). To some extent, this may limit the accuracy of their prognostic assessments. Therefore, this retrospective cohort study aims to identify independent risk factors for 28-day mortality in patients with septic shock. We will construct and validate a highly targeted, superior prognostic prediction model incorporating cardiac function indicators. This model will provide reliable theoretical support and practical clinical tools for early identification of high-risk patients and individualized optimization of treatment strategies.

## 2 Materials and methods

### 2.1 Study design and population

This retrospective, multicenter cohort study enrolled adult patients with septic shock treated in the Intensive Care Unit (ICU), Emergency Intensive Care Unit (EICU), and Neurocritical Care Unit (NCU) of Ningxia Medical University General Hospital, as well as the ICU of Ningxia Medical University Cardiovascular and Cerebrovascular Hospital. Using the hospital’s medical record management system, 226 patients discharged from the General Hospital ICU between September 2021 and October 2024 were identified as the training cohort. 132 patients discharged from the ICU, EICU, NCU, and the ICU of Ningxia Medical University Cardiovascular Hospital between November 2024 and December 2025 and diagnosed with septic shock were used as the validation cohort.

**FIGURE 1:**
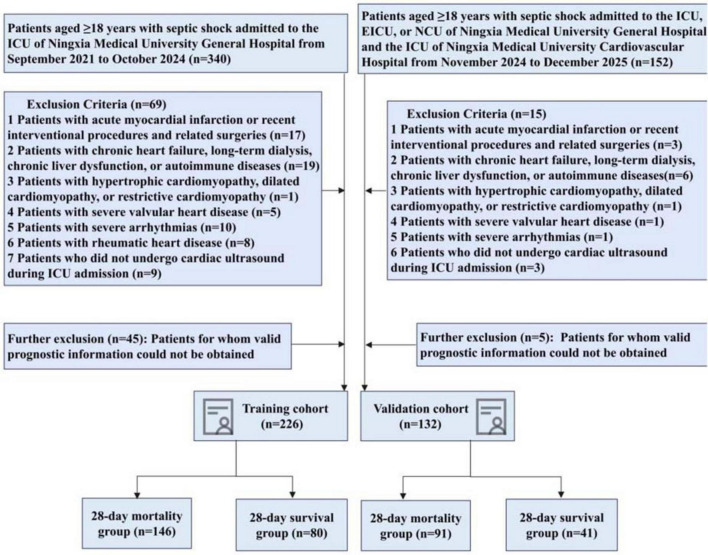
Flowchart of study population screening.

The study population comprised patients diagnosed with septic shock who met the clinical trial screening criteria for sepsis. Inclusion criteria: 1. Compliance with the Sepsis 3.0 diagnostic criteria (1); 2. Age ≥ 18 years. Exclusion criteria: 1. Patients with acute myocardial infarction or recent interventional procedures/related surgeries; 2. Patients with chronic heart failure, long-term dialysis, chronic liver failure, or autoimmune diseases; 3. Patients with hypertrophic cardiomyopathy, dilated cardiomyopathy, or restrictive cardiomyopathy; 4. Patients with severe valvular heart disease; 5. Patients with severe arrhythmias; 6. Patients with rheumatic heart disease; 7. Patients who did not undergo cardiac ultrasound during ICU admission. Based on 28-day survival, the training cohort was divided into a survival group (*n* = 80) and a mortality group (*n* = 146), while the validation cohort comprised a survival group (*n* = 41) and a mortality group (*n* = 91) (Figure 1).

This study was conducted in accordance with the Declaration of Helsinki and has been approved by the Ethics Committee of Ningxia Medical University General Hospital (Approval No.KYLL-2026-0210).

### 2.2 Echocardiographic assessment

This study employed the GE Vivid E9 ultrasound system to acquire transthoracic echocardiography (TTE) data for evaluating cardiac function in patients. Echocardiographic findings were collected for all subjects, with the time of the first TTE during ICU admission serving as the key assessment point for cardiac dysfunction in patients with septic shock. The LVEF result at this time point was selected as the final analysis indicator. TTE examinations were performed at the bedside by certified echocardiography technicians, strictly adhering to imaging standards recommended by the American Society of Echocardiography (11).

### 2.3 Clinical data

Record patient demographics, Acute Physiology and Chronic Health Evaluation (APACHE II) score, and Sequential Organ Failure Assessment (SOFA) score within 24 h of ICU admission. Clinical indicators include heart rate (beats/min), mean arterial pressure (MAP, mmHg), invasive ventilation status, electrocardiogram results, infection site, pathogen identification results, vasoactive drug types, etc. Laboratory test indicators include lactate (Lac, mmol/L), creatinine (Cr, μmol/L), total bilirubin (TB, μmol/L), PaO_2_/FiO_2_ (P/F, Arterial Oxygen Partial Pressure/Fraction of Inspired Oxygen, mmHg/%) cardiac troponin I (cTnI, ng/mL), N-terminal pro-brain natriuretic peptide (NT-proBNP, pg/mL), white blood cells (WBC, 10^9^/L), hemoglobin (HGB, g/L), platelets (PLT, 10^9^/L), and absolute lymphocyte count (LYM, 10^9^/L). All laboratory indicators use the worst value within 24 h.

### 2.4 Definition

septic shock is defined as the presence of sepsis with serum lactate levels > 2 mmol/L (excluding other causes of tissue hypoperfusion) requiring vasopressors to maintain mean arterial pressure ≥ 65 mmHg despite adequate fluid resuscitation (1). Left ventricular systolic dysfunction is defined as an initial echocardiogram after ICU admission showing a LVEF < 50% (cardiac suppression) or LVEF > 70% (cardiac hyperdynamism) (7, 12). Severe arrhythmia is defined as persistent ventricular tachycardia, ventricular fibrillation, third-degree atrioventricular block, or arrhythmias leading to hemodynamic instability (13). Echocardiography is defined as moderate or more severe stenosis or regurgitation in severe valvular heart disease (14). The 28-day mortality rate is defined as death from any cause occurring within 28 days from the initial echocardiogram (15).

### 2.5 Statistical analysis

Statistical analysis was performed using R software, with *P* < 0.05 as the threshold for statistical significance. Descriptive analysis employed the Shapiro–Wilk test to assess normality of continuous data. Normally distributed data were presented as “mean ± standard deviation,” while non-normally distributed data were presented as “median (interquartile range).” Categorical variables were reported as “frequency (percentage).” Intergroup comparisons were performed using Kruskal-Wallis test, chi-square test, or Fisher’s exact test based on variable distribution characteristics and data types. Independent predictors of 28-day mortality in patients with septic shock were identified through univariate and multivariate logistic regression analyses to construct a predictive model. The model’s discriminatory ability was evaluated using receiver operating characteristic (ROC) curves. Model calibration was assessed via the Hosmer-Lemeshow test and calibration curves. Clinical net benefit was evaluated using decision curve analysis (DCA). Model stability was validated through bootstrap resampling (1,000 iterations).

## 3 Result

### 3.1 Demographic characteristics of study populations

In the training cohort, 340 patients with septic shock underwent enrollment assessment. Following exclusion criteria, 69 patients were excluded. An additional 45 patients were excluded due to loss to follow-up. Ultimately, 226 patients were included in the training cohort. In the validation cohort, 15 patients were excluded based on exclusion criteria, and 5 patients were excluded due to loss to follow-up. Ultimately, 132 patients were included in the validation cohort. There were no statistically significant differences in baseline characteristics between the training and validation cohorts (all *P* > 0.05; Table 1).

**TABLE 1:** Baseline characteristics of all patients in the training cohort and validation cohort.

Variable	Level	Training cohort (*n* = 226)	Validation cohort (*n* = 132)	*P*-value
Age (years)		70 (58–77)	70 (57–76.5)	0.119
Gender (%)	Male	152 (67.3)	91 (69.5)	0.754
	Female	74 (32.7)	40 (30.5)	
DM(%)	0	185 (81.9)	95 (72.5)	0.053
	1	41 (18.1)	36 (27.5)	
HTN(%)	0	154 (68.1)	76 (58.0)	0.070
	1	72 (31.9)	55 (42.0)	
COPD(%)	0	210 (92.9)	122 (93.1)	1.000
	1	16 (7.1)	9 (6.9)	
CHD(%)	0	187 (82.7)	98 (74.8)	0.096
	1	39 (17.3)	33 (25.2)	

DM, diabetes mellitus; HTN, hypertension; COPD, chronic obstructive pulmonary disease; CHD, coronary heart disease.

The study population in both the training and validation cohorts consisted primarily of elderly patients, with a median age of 70 years. The training cohort included 152 males (67.3%), while the validation cohort had 91 males (69.5%). The main comorbidities were hypertension, diabetes, coronary heart disease, and chronic obstructive pulmonary disease. No significant differences were observed between the training and validation cohorts in these baseline characteristics (*P* > 0.05).

### 3.2 General characteristics of the training cohort

Left ventricular systolic dysfunction (LVSD, LVEF < 50% or > 70%, *n* = 59) was present in 26.11% of the training cohort, while 73.89% had normal LV systolic function (50% ≤ LVEF ≤ 70%, *n* = 167). The 226 patients were divided into a mortality group (146 cases, 64.6%) and a survival group (80 cases, 35.4%). Based on 28-day survival.

Compared with the survival group, patients in the mortality group had higher age, faster heart rate, lower laboratory values (pH, PaO_2_/FiO_2_, hemoglobin concentration), higher clinical scores (APACHE II, SOFA), increased vasopressor usage (dopamine, dobutamine), and elevated rates of atrial/ventricular arrhythmias and blood lactate levels (all *P* < 0.05). Notably, the incidence of left ventricular systolic dysfunction was significantly higher in the mortality group than in the survival group (30.8% vs. 17.5%, *P* = 0.043) (Table 2).

**TABLE 2:** Comparison of demographic and baseline characteristics between the 28-day mortality group and survival group.

Variable	Total (*n* = 226)	28-day mortality group (*n* = 146)	28-day survival group (*n* = 80)	*P*-value
Age (years)	70 (58–77)	72 (61–79)	64.5 (53–72)	< 0.001***
Gender				0.699
Male(%)	152 (67.3)	52 (68.5)	100 (65.0)	
Female(%)	74 (32.7)	28 (31.5)	46 (35.0)	
DM(%)	41 (18.1)	28 (19.2)	13 (16.2)	0.715
HTN(%)	72 (31.9)	50 (34.2)	22 (27.5)	0.373
COPD(%)	16 (7.1)	10 (6.8)	6 (7.5)	1.000
CHD(%)	39 (17.3)	27 (18.5)	12 (15.0)	0.631
HR(bpm)	109 (94–127)	110 (96–130)	102 (91.5–117.5)	0.014*
MAP (mmHg)	85.50 (74.00–99.70)	83.30 (73.30–98.53)	89.50 (76.60–101.03)	0.285
PH	7.38 (7.29–7.43)	7.36 (7.27–7.41)	7.41 (7.35–7.46)	< 0.001***
PaO_2_ (mmHg)	81.60 (70.60–111.75)	81.40 (68.85–111.75)	83.10 (73.30–112.19)	0.321
PaCO_2_ (mmHg)	37.65 (31.90–46.03)	38.03 (32.35–46.77)	37.15 (31.57–42.42)	0.166
P/F (mmHg/%)	178.50 (130.00–235.68)	169.00 (120.75–229.75)	196.00 (154.00–252.00)	0.008**
Lac (mmol/L)	2.40 (1.50–4.57)	2.80 (1.80–5.57)	1.80 (1.10–2.69)	< 0.001***
cTnI (ng/mL)	0.14 (0.04–0.67)	0.16 (0.04–0.91)	0.12 (0.05–0.33)	0.341
Nt-proBNP (pg/mL)	4740.23 (1425.57–12551.05)	5358.95 (1533.60–13629.56)	3946.10 (1210.45–8642.81)	0.089
Cr (μmol/L/L)	46.65 (1.38–109.53)	49.65 (1.16–120.64)	43.05 (1.63–94.60)	0.805
TBil (μmol/L/L)	27.85 (16.33–58.25)	28.70 (17.10–57.25)	24.45 (14.47–58.57)	0.289
WBC (10^9^/L)	10.23 (6.14–15.93)	10.27 (5.95–15.74)	10.07 (7.06–16.53)	0.703
Hb (g/L)	102.50 (86.25–120.00)	105.00 (88.25–127.75)	95.50 (83.75–110.25)	0.019*
PLT (10^9^/L)	116.00 (59.25–210.75)	115.00 (54.50–198.75)	116.00 (68.75–213.50)	0.566
LYM (10^9^/L)	0.77 (0.37–1.23)	0.73 (0.34–1.21)	0.90 (0.45–1.24)	0.179
Pulmonary infection(%)	195 (86.3)	121 (82.9)	74 (92.5)	0.071
Abdominal infection(%)	93 (41.2)	64 (43.8)	29 (36.2)	0.334
Hematogenous infection(%)	67 (29.7)	43 (29.5)	24 (30.0)	1.000
Urinary infection(%)	18 (8.0)	8 (5.5)	10 (12.5)	0.108
Gram positive bacteria(%)	156 (69.0)	94 (64.4)	62 (77.5)	0.059
Gram-negative bacteria(%)	153 (67.7)	98 (67.1)	55 (68.8)	0.919
Fungus(%)	71 (31.4)	44 (30.1)	27 (33.8)	0.682
Virus(%)	13 (5.8)	5 (3.4)	8 (10.0)	0.069
LVEF(%)	61.91 (52.51–64.95)	62.03 (50.82–65.36)	61.90 (57.92–64.36)	0.872
LVSD(%)	59 (26.1)	45 (30.8)	14 (17.5)	0.043*
LVDD(%)	165 (73.0)	103 (70.5)	62 (77.5)	0.333
Norepinephrine(%)	222 (98.2)	144 (98.6)	78 (97.5)	0.616
Dobutamine(%)	31 (13.7)	26 (17.8)	5 (6.2)	0.027*
Dopamine(%)	81 (35.8)	63 (43.2)	18 (22.5)	0.003**
Invasive ventilation(%)	192 (85.0)	128 (87.7)	64 (80.0)	0.178
Atrial arrhythmia(%)	50 (22.1)	43 (29.5)	7 (8.8)	0.001**
Ventricular arrhythmia(%)	20 (8.9)	18 (12.3)	2 (2.5)	0.025*
Borderline arrhythmia(%)	5 (2.2)	4 (2.7)	1 (1.2)	0.658
APACHE II score	21.15 ± 6.79	22.38 ± 6.79	18.90 ± 6.23	< 0.001***
SOFA score	11.00 (9.00–13.75)	12.00 (9.00–14.00)	10.00 (8.00–12.00)	0.003**

DM, diabetes mellitus; HTN, hypertension; COPD, chronic obstructive pulmonary disease; CHD, coronary heart disease, HR, heart rate; MAP, mean arterial pressure; PaO_2_, partial pressure of arterial oxygen; PaCO_2_, partial pressure of arterial carbon dioxide; P/F, PaO_2_/FiO_2_; Lac, lactate; cTnI, cardiac troponin I; NT-proBNP, N-terminal pro-brain natriuretic peptide; Cr, creatinine; TBil, total bilirubin; WBC, white blood cell count; Hb, hemoglobin; PLT, platelet count; LYM, lymphocyte count; LVEF, left ventricular ejection fraction; LVSD, left ventricular systolic dysfunction; LVDD, left ventricular diastolic dysfunction; APACHE II, Acute Physiology and Chronic Health Evaluation II score; SOFA, Sequential Organ Failure Assessment score.

*P < 0.05, **P < 0.01, ***P < 0.001.

### 3.3 Development and assessment of the nomogram

Univariate logistic analyses incorporating laboratory test indicators, clinical indicators, and echocardiographic indicators identified potential factors associated with 28-day prognosis outcome (all *P* < 0.05): pH, gender, PaO_2_/FiO_2_, hemoglobin concentration, age, APACHE II score, SOFA score, diabetes, lactate, hypertension, LVSD, dopamine use, and atrial arrhythmia (Table 3). Statistically significant variables from univariate analysis were entered into a multivariate logistic regression model for stepwise selection (using backward stepwise method, with *P* > 0.05 as the exclusion criteria). Decreased pH, atrial arrhythmia, dopamine use, reduced PaO_2_/FiO_2_, and LVSD were identified as independent predictors of 28-day mortality. Based on these predictors, a predictive model for 28-day mortality in patients with septic shock was constructed (Table 4 and Supplementary Figure 1).

**TABLE 3:** Univariate analysis of 28-day mortality in patients with septic shock.

Variable	OR	95%CI	*P*-value
PH	0.100	0.100–0.100	< 0.001***
Gender	0.838	0.471–1.502	0.549
P/F	0.995	0.992–0.999	0.006**
Hb	1.015	1.003–1.027	0.013*
Age	1.035	1.016–1.056	< 0.001***
APACHE II score	1.083	1.037–1.134	< 0.001***
SOFA score	1.112	1.028–1.206	0.009**
DM	1.205	0.594–2.550	0.614
Lac	1.23	1.102–1.405	< 0.001***
HTN	1.349	0.747–2.487	0.326
LVSD	2.069	1.074–4.184	0.035*
Dopamine	2.768	1.501–5.308	0.002**
Atrial arrhythmia	4.294	1.934–10.000	< 0.001***

P/F, PaO_2_/FiO_2_; Hb, hemoglobin; APACHE II score, Acute Physiology and Chronic Health Evaluation II score; SOFA score, Sequential Organ Failure Assessment score; DM, diabetes mellitus; Lac, lactate; HTN, hypertension; LVSD, left ventricular systolic dysfunction.

*P < 0.05, **P < 0.01, ***P < 0.001.

**TABLE 4:** Multivariate logistic regression analysis of independent risk factors.

Variable	OR	95%CI	*P*-value
Atrial arrhythmia	5.119	2.152–13.871	< 0.001***
Dopamine	2.452	1.264–4.916	0.009**
P/F	0.995	0.991–0.999	0.021*
LVSD	2.643	1.252–5.888	0.013*
PH	0.005	0.000–0.130	0.002**

P/F, PaO_2_/FiO_2_; LVSD, left ventricular systolic dysfunction.

*P < 0.05, **P < 0.01, ***P < 0.001.

**FIGURE 2:**
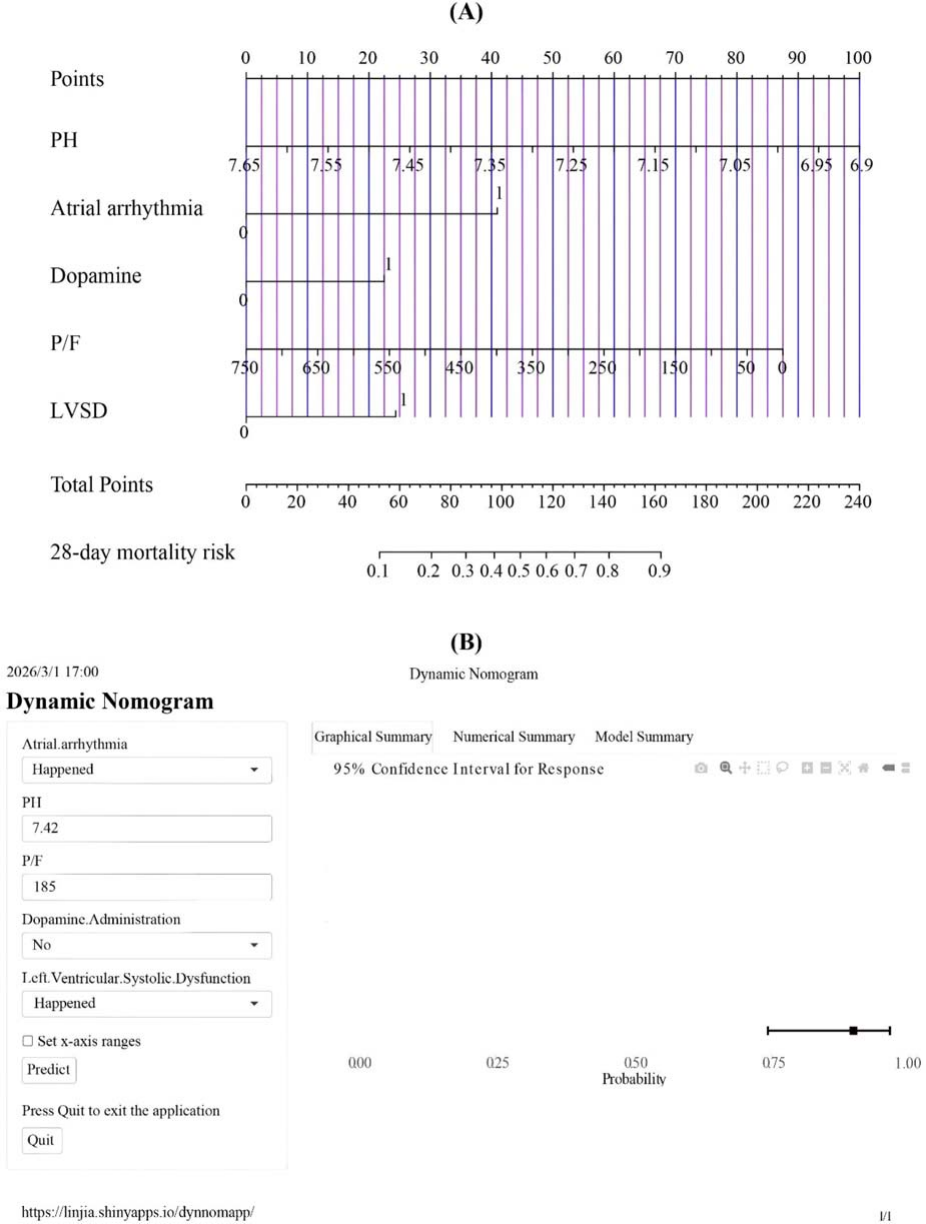
**(A)** Nomogram for the 28-day mortality risk of septic shock. **(B)** The online web-based calculator for predicting the 28-day mortality risk of septic shock. **(A,B)** The nomogram incorporates the following predictors: Atrial arrhythmia, Dopamine, PaO_2_/FiO_2_, LVSD, and PH. Each predictor is assigned a score on a specific point scale. To use the nomogram, clinicians first locate the patient’s value for each predictor on the corresponding axis. Then, the points for each predictor are summed to determine the total score, which is subsequently converted into a probability of 28-day mortality of septic shock. P/F, PaO_2_/FiO_2_; LVSD, left ventricular systolic dysfunction.

A nomogram was constructed to predict 28-day mortality in patients with septic shock based on independent predictors (Figure 2A). This nomogram assigns scores based on the patients’ pH level, presence of atrial arrhythmia, dopamine use, PaO_2_/FiO_2_, and occurrence of left ventricular systolic dysfunction. The sum of individual scores yields a total score, enabling an intuitive prediction of the patient’s 28-day mortality probability. To dynamically assess changes in the nomogram, we developed a web-based dynamic nomogram calculator^1^ (Figure 2B). Clinicians need only input the patient’s five aforementioned indicators, and the calculator automatically performs score conversion and mortality probability prediction, facilitating rapid result retrieval and intuitive observation of data trends.

### 3.4 Model discrimination

**FIGURE 3:**
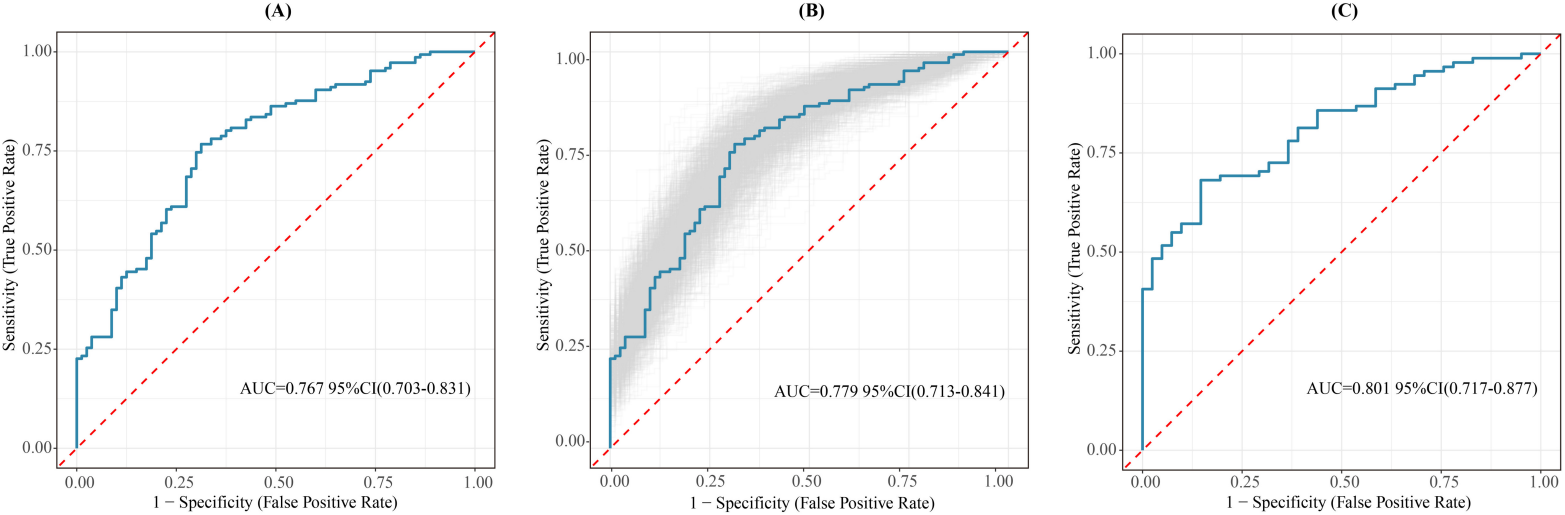
Receiver operating characteristic (ROC) curves. **(A)** Training cohort. **(B)** Training cohort with 1,000 bootstrap resamples. **(C)** Validation cohort. The Y-axis represents sensitivity (true positive rate), and the X-axis represents 1- specificity (false positive rate). The blue stepwise curve shows the ROC curve of the prediction model. The red dashed diagonal line indicates the reference line corresponding to random discrimination (AUC = 0.5). The gray shaded area represents the ROC curves obtained from 1,000 resampling attempts.

In the training cohort, the receiver operating characteristic (ROC) analysis of the predictive model for 28-day mortality in patients with septic shock revealed an area under the curve (AUC) of 0.767 (95% CI: 0.703–0.831), indicating the model possessed good discriminatory ability (Figure 3A).

To further assess the stability of the model’s discriminatory ability, internal validation was performed using 1000 Bootstrap resamples. Results showed a mean AUC of 0.779 (95% CI: 0.713–0.841). This predictive model exhibits high discriminatory ability with minimal performance fluctuation across repeated sampling, demonstrating good stability (Figure 3B).

When evaluating the model’s predictive efficacy for 28-day mortality outcomes in the validation cohort, the AUC reached 0.801 (95% CI: 0.717–0.877), exceeding the training cohort’s AUC (0.767). The model maintained robust discriminatory ability in external validation while showing a slight improvement in predictive performance (Figure 3C).

**FIGURE 4:**
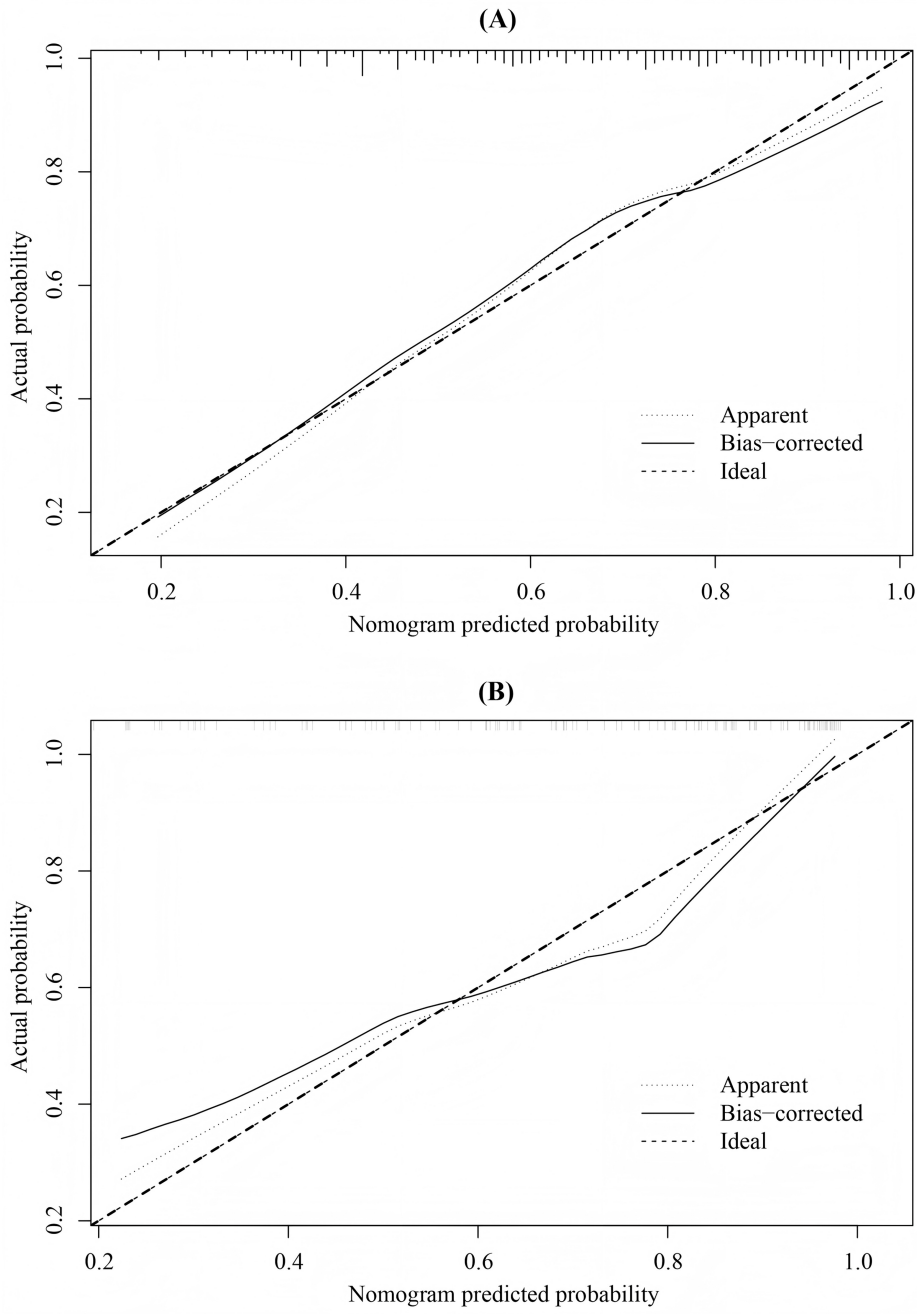
Calibration curves for mortality prediction model. **(A)** Training cohort. **(B)** Validation cohort. The X-axis represents the nomogram-predicted probability of 28-day mortality, and the Y-axis represents the observed actual probability of 28-day mortality. The dotted line (Apparent) indicates the apparent calibration performance in the cohort, the solid line (Bias-corrected) shows the bias-corrected calibration performance after 1,000 bootstrap resamples.

**FIGURE 5:**
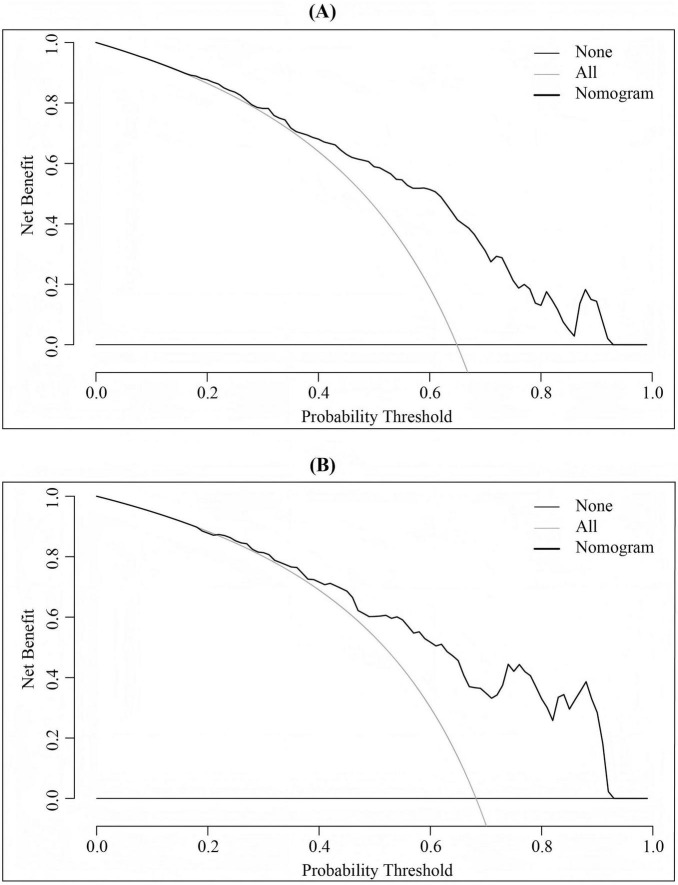
Decision curve analysis in prediction. **(A)** Training cohort. **(B)** Validation cohort. The X-axis represents the threshold probability of 28-day mortality, and the Y-axis represents the net benefit of the model. The solid black line (Predictors) shows the net benefit of using the nomogram to predict 28-day mortality. The gray line (All) indicates the net benefit of assuming all patients will experience 28-day mortality, while the horizontal black line (None) represents the net benefit of assuming no patients will experience 28-day mortality.

In the training cohort, the calibration curve of the predictive model was close to the ideal diagonal line. The Hosmer-Lemeshow test yielded a *P*-value of 0.476, indicating a good agreement between model predictions and observed outcomes (Figure 4A). The Brier score was 0.180, which indicated that the model had acceptable overall predictive accuracy and reliable probabilistic calibration. Decision curve analysis (DCA) demonstrated that across a broad range of threshold probabilities (0.05–0.85), the predictive model provided a higher net benefit compared to either “treat all” or “treat none” strategies (Figure 5A). For example, at a threshold of 0.4, the model’s net benefit is approximately 0.08 higher than the “treat all” strategy, indicating its effectiveness in reducing overtreatment and guiding the optimization of individualized treatment strategies, thus demonstrating good clinical practicality.

In the validation cohort, the calibration curve of the predictive model similarly approached the ideal diagonal line, with a Hosmer-Lemeshow test *P*-value of 0.432, indicating acceptable agreement between the model-predicted 28-day mortality outcomes and actual events (Figure 4B). Meanwhile, the Brier score was 0.164, showing improved predictive performance and probabilistic calibration relative to the training cohort. DCA also demonstrated favorable net benefit for the predictive model in the validation cohort (Figure 5B).

Collectively, these results confirm that the logistic regression model developed in this study exhibits excellent calibration performance and provides reliable prediction accuracy for 28-day mortality risk.

### 3.5 Comparison of predictive models and APACHE II score for 28-Day prognosis

**FIGURE 6:**
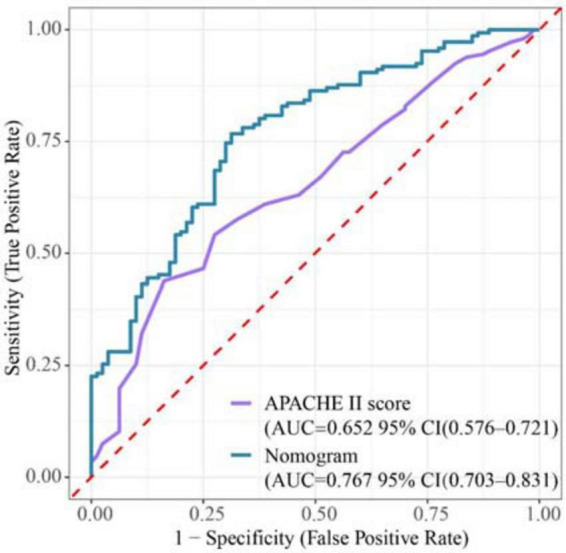
ROC comparison of APACHE II score and predictive model. APACHE II score = Acute Physiology and Chronic Health Evaluation II. The Y-axis represents sensitivity (true positive rate), and the X-axis represents 1- specificity (false positive rate). The blue stepwise curve shows the ROC curve of the prediction model. The purple stepwise curve shows the ROC curve of the APACHE II score. The red dashed diagonal line indicates the reference line corresponding to random discrimination (AUC = 0.5).

ROC curve analysis revealed that the predictive model developed in this study (AUC = 0.767) significantly outperformed the APACHE II score [AUC = 0.652, 95% CI (0.576–0.721), *P* = 0.006] in distinguishing 28-day mortality outcomes among patients with septic shock. The prediction model’s curve remained entirely above both the APACHE II score curve and the random guessing diagonal (AUC = 0.5), indicating that at any false positive rate level, this model achieved a higher true positive rate and could more accurately identify patients at high risk of death. This suggests its better efficacy in assessing prognosis among patients with septic shock (Figure 6).

## 4 Discussion

Accurately identifying septic shock patients at high risk of mortality and implementing individualized interventions are key to improving outcomes (5, 16).. Previous studies have confirmed that left ventricular systolic dysfunction is closely associated with poor outcomes in patients with septic shock (17, 18). However, widely used scoring systems for diagnosing and predicting outcomes in septic shock patients, such as the SOFA score and APACHE II score, do not include left ventricular systolic function indicators. This may indicate that the current assessment of prognosis for septic shock is not complete (10, 19). Therefore, this study centered on the core question of “the association between left ventricular systolic dysfunction and prognosis in patients with septic shock.” Through a series of studies (including the analysis of clinical indicators, the construction and validation of predictive models, and the evaluation of clinical value), the important roles of left ventricular systolic dysfunction, atrial arrhythmia, decreased pH, dopamine use, and reduced PaO_2_/FiO_2_ in predicting 28-day mortality in septic shock have been clarified. At present, a prognostic prediction model with high accuracy and applicability has been established (20). The model demonstrates good discrimination, calibration, and clinical net benefit, and its predictive performance is significantly superior to the traditional APACHE II score. Our study found that including left ventricular systolic dysfunction (defined as LVEF < 50% or > 70%) in risk assessment can optimize prognostic prediction performance, providing a simple and practical clinical tool for early identification of high-risk patients and guiding individualized critical care.

### 4.1 Clinical indicator differences and pathophysiological mechanisms

Results revealed significant differences in multiple clinical indicators between the mortality and survival groups. These disparities not only reflect disease severity but also provide crucial clues for prognosis assessment. In this study, the 28-day mortality rate for patients with septic shock was 64.6%, higher than the mortality rates reported in most studies on septic shock. This difference primarily stems from the high-risk characteristics of the enrolled population: only patients with septic shock who were admitted to and discharged from the ICU were included, a group that often experiences more severe multi-organ dysfunction, higher incidence of cardiac abnormalities, and increased baseline risk of death. Additionally, to focus on early cardiac function changes in septic shock, the study excluded patients with milder conditions who did not undergo cardiac ultrasound within 24 h, which may have further enriched the cohort with severe cases and contributed to the overall higher mortality rate. Although selecting this high-risk population may overestimate mortality and limit the generalizability of the results, constructing a prognostic model for this high-risk subgroup has significant clinical value. The mortality group exhibited marked abnormalities across multidimensional pathophysiological parameters: advanced age, tachycardia, acid-base imbalance (decreased pH, elevated PaCO_2_), impaired oxygenation (reduced PaO_2_/FiO_2_), elevated lactate, decreased hemoglobin, and significantly higher rates of left ventricular systolic dysfunction (30.8% vs. 17.5%, *P* = 0.043) and positive inotropic agent use. These differences not only validate previous research concluding that advanced age, tissue hypoxia, and cardiac dysfunction are poor prognostic risk factors for septic shock (21, 22), but also more clearly map the pathophysiological chain of “inflammatory response—organ dysfunction—circulatory failure—death” (16, 23–25).

Left ventricular systolic dysfunction (LVSD) is a key factor in the progression of septic shock and is also the central focus of this article. From a pathophysiological perspective, LVSD caused by septic shock is not merely a decline in cardiac function. Instead, it is the result of a combination of systemic inflammatory storm-induced disturbances in myocardial energy metabolism, abnormal calcium regulation, reduced β-receptor sensitivity, and overactivation of neurohumoral systems, which subsequently leads to inadequate tissue perfusion, oxygen supply-demand imbalance, and ultimately multi-organ failure (25, 26). In our study, we found that LVSD is an independent predictor of 28-day mortality in septic shock, providing prognostic information independently of factors such as age, lactate levels, and APACHE II scores. Its high incidence in the deceased group further underscores its core value in prognostic assessment. This study also further confirms that incorporating LVSD into predictive models provides tangible and quantifiable additional value, significantly enhancing the effectiveness of prognostic prediction and compensating for the limitations of traditional scoring systems in assessing cardiac function.

Furthermore, the significantly elevated incidence of arrhythmias and higher APACHE II and SOFA scores in the mortality group corroborate the prognostic predictive significance of these indicators from the perspectives of organ functional stability and overall disease severity, laying a solid foundation for subsequent variable selection in predictive models (14, 19).

### 4.2 Interpretation of core predictive model indicators

Previous studies have confirmed the association between left ventricular systolic dysfunction and mortality in patients with septic shock (17, 18), but most investigations have excluded hyperkinetic left ventricular status (LVEF > 70%) from the assessment of left ventricular systolic impairment (7). One of the innovations of the study lies in incorporating high-dynamic-state LV into the assessment. Results revealed that the incidence of left ventricular systolic dysfunction (LVEF < 50% or LVEF > 70%) was significantly higher in the mortality group than in the survival group. This suggests that overall impairment of left ventricular systolic function—whether manifested as hypodynamic state with reduced ejection fraction or hyperdynamic state with elevated ejection fraction—is associated with poor prognosis. This finding breaks through the traditional limitation of focusing solely on low-ejection-fraction cardiac dysfunction, and it emphasizes that abnormal myocardial contractility is a key factor influencing prognosis. Hyperdynamic left ventricular dysfunction may represent a compensatory effort to maintain circulation during severe infection and inflammatory response. However, this compensation is limited and unsustainable, potentially progressing to hypodynamic states with disease advancement and ultimately leading to circulatory failure. Thus, early recognition of this state is crucial for comprehensive assessment of disease severity and prognosis (16).

Multivariate logistic regression analysis further confirmed that left ventricular systolic dysfunction is an independent predictor of 28-day mortality in patients with septic shock (OR = 2.643, 95% CI: 1.252–5.888, *P* = 0.013). Its predictive value remains independent of traditional factors such as age and infection site, providing unique prognostic information that cannot be replaced by conventional indicators such as heart rate, blood pressure, lactate, and troponin. It provides a new critical reference indicator for clinical prognosis assessment. In clinical risk stratification, LVSD can serve as a quantifiable functional indicator, enhancing the accuracy of predicting 28-day mortality prognosis in patients with septic shock. This addresses the deficiency of existing scoring systems (such as APACHE II scoring) in capturing changes in cardiac function. Therefore, LVSD possesses independent, stable, and incremental prognostic value, making it a core variable in this prognostic model. Additionally, other core indicators in the model each demonstrated distinct prognostic significance: Atrial arrhythmia was the predictor with the highest odds ratio (OR = 5.119, 95% CI: 2.152–13.871, *P* < 0.001). By reducing cardiac output, increasing myocardial oxygen consumption, and elevating thromboembolic risk, it exacerbates the already compromised circulatory burden in septic shock patients, accelerating disease progression (27, 28). Dopamine use (OR = 2.452, 95% CI: 1.264–4.916, *P* = 0.009) directly reflects the severity of circulatory failure in septic shock patients, indicating poor initial fluid resuscitation response and serving as an objective marker of critical illness. Guidelines for the diagnosis and treatment of infectious shock recommend norepinephrine as the first-line drug for resuscitation (16). During data collection, we also recorded the use of norepinephrine. However, statistical analysis showed that the *P*-value was greater than 0.05, which may be related to the fact that most patients with infectious shock were administered norepinephrine for blood pressure support. Unlike using norepinephrine alone to raise blood pressure, dopamine is commonly used in refractory circulatory failure and other critical conditions, where positive inotropic agents are required to further enhance cardiac function. Therefore, the administration of dopamine can serve as an objective indicator reflecting the severity of circulatory failure and underlying diseases. This clinical feature further corroborates the central driving role of cardiac dysfunction in the progression of septic shock (29, 30). A reduced PaO_2_/FiO_2_ (OR = 0.995, 95% CI: 0.991–0.999, *P* = 0.021) is closely associated with ARDS and MODS in patients with septic shock. A decrease in PaO_2_/FiO_2_ in patients with septic shock indicates severe acute respiratory dysfunction, increased intrapulmonary shunt, and impaired tissue oxygen delivery, which directly exacerbates multiple organ failure and increases the risk of death (31). This is consistent with previous research findings that the PaO_2_/FiO_2_ can serve as an independent predictor of short-term prognosis in septic shock (32). As a core indicator of acid-base balance, decreased pH (OR = 0.005, 95% CI: 0.000–0.130, *P* = 0.002) signifies acidosis that inhibits myocardial contractility and reduces vascular responsiveness to active drugs, serving as a strong signal of poor prognosis (16, 33). This study analyzes both pH value and PaO_2_/FiO_2_ as continuous variables. The lower the values of these two parameters, the higher the risk of patient mortality. This association is clearly presented in the nomogram and is consistent with the conclusions of existing studies.

The combined application of these core indicators established a comprehensive prognostic assessment system for patients with septic shock, encompassing cardiac function, circulatory status, respiratory function, and acid-base balance. These indicators reveal mortality risk from distinct pathophysiological pathways, and their synergistic integration enables predictive models to more accurately identify high-risk populations.

### 4.3 Clinical utility of the predictive model

Through univariate and multivariate logistic regression analysis, this study identified five independent predictors—LVSD, decreased pH, atrial arrhythmia, dopamine use, and reduced PaO_2_/FiO_2_—to construct a 28-day mortality prediction model for patients with septic shock. ROC curve analysis revealed an AUC of 0.767, indicating good risk discrimination capability. The model demonstrated excellent calibration in both training and validation cohorts (Hosmer-Lemeshow test *P*-values: 0.476 and 0.432, respectively) and significant clinical net benefit (DCA curves showed higher net benefit across a broad range of threshold probabilities). Further validating its reliability and value in guiding clinical decision-making. The nomogram derived from this model translates quantitative weights of predictive factors into a visual scoring system, enabling clinicians to rapidly and intuitively estimate 28-day mortality risk based on early clinical indicators, significantly enhancing the model’s practical utility.

A clinical practice scenario example is as follows: For a patient with septic shock complicated by LVSD, presenting with a pH of 7.45, an PaO_2_/FiO_2_ of 450, concomitant atrial arrhythmia, and no dopamine therapy, the nomogram assessment indicates a 28-day mortality risk of 68.7%. This result clearly indicates the need for early initiation of individualized interventions targeting cardiac function in such patients to reduce the risk of poor outcomes.

This convenient risk assessment tool is particularly suitable for use during the initial hospital admission or when clinical status changes in septic shock patients. It helps clinicians rapidly identify individuals at high potential mortality risk, providing crucial evidence for formulating early intervention strategies. The development of a web-based dynamic nomogram calculator addresses the limitation of traditional nomograms in dynamic assessment. By enabling real-time input of patient clinical data, it facilitates dynamic risk updates and tracking, offering immediate reference for adjusting treatment plans during clinical status changes.

### 4.4 Comparative predictive performance between the prediction model and traditional scores

The APACHE II score remains a widely used prognostic assessment tool for septic shock in clinical practice. It incorporates 12 parameters—including age, mean arterial pressure, body temperature, heart rate, respiratory rate, arterial oxygen partial pressure, arterial pH, serum sodium, potassium, creatinine, hematocrit, white blood cell count, and Glasgow Coma Scale (GCS) score. A higher score indicates more severe illness and poorer prognosis (6, 7). Results from this study demonstrated that the APACHE II score was significantly higher in the mortality group compared to the survival group (22.38 ± 6.79 vs. 18.90 ± 6.23, *P* < 0.001), confirming its value in assessing prognosis for patients with septic shock. However, despite incorporating multiple acute physiological and chronic health indicators, the APACHE II score excludes core parameters reflecting cardiac function status—such as left ventricular ejection fraction, myocardial injury markers, or diagnostic criteria for cardiac dysfunction—beyond heart rate. This omission may lead to inadequate assessment of cardiac function in septic shock patients.

The predictive model developed in this study addresses these limitations by identifying left ventricular systolic dysfunction as an independent risk factor for 28-day mortality in septic shock. It combines this key indicator with readily accessible clinical parameters—decreased pH, atrial arrhythmia, dopamine use, and reduced PaO_2_/FiO_2_—to establish a model specifically focused on predicting 28-day mortality in septic shock patients with left ventricular systolic dysfunction. ROC curve analysis revealed an AUC of 0.767 for this model, significantly higher than the APACHE II score (AUC = 0.652, 95% CI: 0.576–0.721, *P* = 0.006). It may demonstrate particular advantage in prognostic assessment for the subgroup of patients with concomitant left ventricular systolic dysfunction.

Furthermore, this model visually presents individualized 28-day mortality risk predictions through the nomogram, offering simplicity and facilitating rapid clinical decision-making. Compared to the cumbersome multi-parameter calculations of the APACHE II score, it demonstrates greater clinical utility. Therefore, the predictive model developed in this study serves as a valuable supplement to the APACHE II score, providing a new effective tool for precise prognosis assessment and risk stratification in patients with septic shock.

This predictive model integrates readily accessible physiological indicators and clinical events to achieve personalized, precise prediction of 28-day mortality risk in patients with septic shock. Combined with its good discrimination, calibration, stability, and significant net clinical benefit, the model demonstrates potential for translation from research findings into a clinical tool. It holds promise as a practical adjunct for clinicians to identify high-risk patients, optimize treatment decisions, and improve patient outcomes.

### 4.5 Study limitations

First, although we collected indicators such as whether mechanical ventilation was used, the site of infection, and the results of infectious pathogens to minimize the impact of confounding factors, and adjusted for some confounding interference through multivariate logistic regression, we were unable to completely control for various clinical heterogeneities. These include differences in mechanical ventilation strategies, timing of drug interventions, individual inflammatory status, and genetic polymorphisms, which may affect the accuracy of the study results. Second, the study included patients with septic shock who were admitted to and discharged from the intensive care units of tertiary hospitals. These patients may have more severe conditions, and those who did not undergo echocardiography within 24 h of admission were excluded, leading to imbalance in baseline disease severity that may overestimate the predictive performance of the model. Third, the study focused on indicators related to left ventricular systolic dysfunction and did not include parameters related to right ventricular structure and function, nor did it conduct dynamic assessments of cardiac function changes. Lastly, the study has limitations regarding region and population, as the enrolled patients were relatively homogeneous in terms of ethnicity, regional environment, and medical accessibility.

Future studies could further expand the sample size across multiple centers and regions, include cases from different levels of medical institutions, broaden the scope of enrolled populations, and reduce biases caused by regional, medical level, and population differences. Comprehensive refinement of clinical intervention indicators, standardization of mechanical ventilation strategies and timing of drug interventions, and integration of potential confounding factors such as individual inflammatory levels and genetic characteristics could further optimize the model. At the same time, improving the cardiac function assessment system by including parameters related to right ventricular function and conducting dynamic temporal monitoring of cardiac function could allow deeper exploration of cardiac function evolution. In addition, prospective cohort studies with extended follow-up can be conducted to validate the external applicability and long-term predictive value of the model, promoting better clinical application of research findings for risk assessment and personalized treatment of patients with septic shock.

## 5 Conclusion

This study developed a prognostic assessment model for patients with septic shock. Based on univariate and multivariate logistic regression analyses, five independent predictors were identified: left ventricular systolic dysfunction, decreased pH, atrial arrhythmia, dopamine use, and reduced PaO_2_/FiO_2_. A 28-day mortality risk prediction model focusing on left ventricular systolic dysfunction was constructed and subjected to internal and external validation. The model demonstrated robust predictive performance in both training and validation cohorts, outperforming the traditional APACHE II score (0.767 vs. 0.652). It exhibits precision, practicality, good calibration, and stability, holding potential for clinical translation to aid in early risk identification and treatment decision-making for patients with septic shock.

## Data Availability

The raw data supporting the conclusions of this article will be made available by the authors, without undue reservation.

## Footnotes

1
https://linjia.shinyapps.io/dynnomapp/

## Supplementary material

Supplementary Figure 1 Univariate and multivariate logistic regression analysis forest plots. **(A)** Univariate analysis of 28-day mortality in patients with septic shock. **(B)** Multivariate logistic regression analysis of independent risk factors.
