# Supplementary figures and images for: Establishment and internal-external validation of a 28-day mortality prediction model for septic shock patients with left ventricular systolic dysfunction
Source: Front Med (Lausanne). 2026 Jun 30;13:1841230. doi: 10.3389/fmed.2026.1841230 (PMC13365332; doi:10.3389/fmed.2026.1841230)

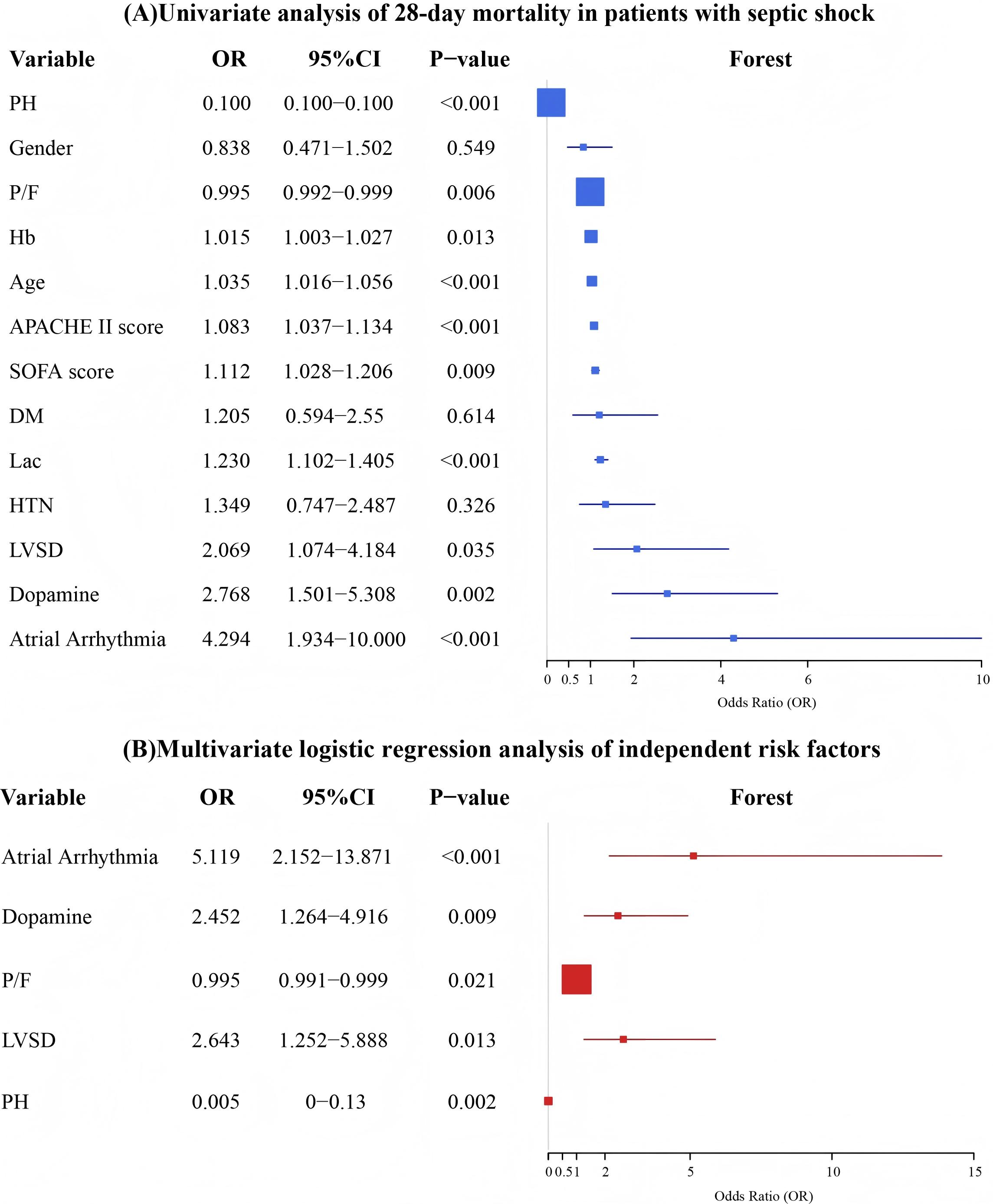

Supplement: Supplementary Figure 1 — Univariate and multivariate logistic regression analysis forest plots. (A) Univariate analysis of 28-day mortality in patients with septic shock. (B) Multivariate logistic regression analysis of independent risk factors. [file Image_1.JPEG]
